# NsrR Represses σ^E^-Dependent Small RNAs and Interacts with RpoE via a Noncanonical Mechanism in *Escherichia coli*

**DOI:** 10.3390/ijms26136318

**Published:** 2025-06-30

**Authors:** Joseph I. Aubee, Jalisa Nurse, Dale Lewis, Chin-Hsien Tai, Karl M. Thompson

**Affiliations:** 1 Department of Microbiology, College of Medicine, Howard University, Washington, DC 20059, USA; jayaub1@outlook.com; 2 Department of Biology, Howard University, Washington, DC 20059, USA; jalisat.nurse@gmail.com; 3 Laboratory of Molecular Biology, National Cancer Institute, National Institutes of Health, Bethesda, MD 20892, USA; lewisdal@mail.nih.gov (D.L.); taic@mail.nih.gov (C.-H.T.)

**Keywords:** small RNA, RpoE, nitric oxide, envelope stress

## Abstract

The envelope stress response in *Escherichia coli* is primarily governed by the sigma factor RpoE (σ^E^), which activates protective genes upon membrane perturbation. Under non-stress conditions, σ^E^ is sequestered by its anti-sigma factor RseA. In this study, we identify an unexpected role for the nitric-oxide-sensing repressor NsrR in dampening σ^E^ activity and repressing σ^E^-dependent small RNAs, including *rybB*, *micA*, and *micL*. Overexpression of *nsrR* represses transcription from σ^E^-dependent promoters and phenocopies σ^E^ inactivation, resulting in filamentous morphology and growth defects. Conversely, Δ*nsrR* de-represses σ^E^ targets, with additive effects in *rseA* mutants—supporting an RseA-independent regulatory role. Time-course analysis shows NsrR represses σ^E^ activity, with kinetics comparable to those of RseA. While in vitro assays failed to detect robust NsrR binding to σ^E^ target promoters, NsrR directly interacts with σ^E^ in bacterial two-hybrid assays. Structural modeling using AlphaFold3 supports a plausible NsrR–RpoE interaction interface. These findings suggest that NsrR functions as a noncanonical anti-sigma-like modulator of σ^E^, integrating redox and envelope stress signals to maintain membrane homeostasis.

## 1. Introduction

RpoE (σ^E^) is an extracytoplasmic function (ECF) sigma factor that governs the envelope stress response in *Escherichia coli* and many other Gram-negative bacteria [1,2,3]. Under non-stress conditions, RpoE is sequestered at the inner membrane by direct binding to its anti-sigma factor (RseA) [4,5]. Activation of the σ^E^ regulon requires the proteolytic degradation of RseA, a process triggered by envelope stress signals, such as misfolded or accumulating outer membrane proteins (OMPs) [2,6,7,8]. These signals initiate a regulated intramembrane proteolysis (RIP) cascade, in which DegS and RseP sequentially cleave RseA, the anti-sigma factor that sequesters σ^E^ to the inner membrane, followed by ClpXP-mediated degradation of the remaining cytoplasmic fragment [4,6,9,10,11]. This cascade releases RpoE to initiate the transcription of target genes involved in maintaining envelope integrity, including chaperones, proteases, and small regulatory RNAs (sRNAs) [12,13,14,15,16,17,18,19,20,21,22].

Among the most critical outputs of the σ^E^ response are sRNAs that downregulate OMP synthesis, thereby reducing the burden on the envelope and helping to restore homeostasis [13,15,23,24]. Key members of this group include MicA, MicL, and RybB, all of which repress the translation of OMP-encoding mRNAs [15,18,25,26,27]. These sRNAs function in negative feedback loops that limit RpoE activation and fine-tune the envelope stress response. RybB, an 80-nucleotide sRNA under the direct control of RpoE, has been shown to target multiple OMPs and participate in ppGpp-mediated stress adaptation [13,15,20,24,28,29,30,31,32].

During a genetic screen for novel repressors of *rybB*, we identified a multicopy clone containing *nsrR*, a nitric oxide (NO)-responsive transcriptional repressor [15,33,34]. Overexpression of *nsrR* was sufficient to suppress both the expression of *rpoE* and *rybB*, even in the absence of RseA, suggesting an RseA-independent mechanism of repression [15]. These findings raised the possibility that NsrR may influence the σ^E^ response through an uncharacterized regulatory interaction. NsrR is a conserved NO-sensing transcriptional repressor that plays central roles in oxidative stress defense and NO detoxification in diverse bacterial species [33,34]. Chromatin immunoprecipitation and microarray analyses (ChIP-chip) have identified dozens of direct NsrR targets in *E. coli*, including genes involved in motility and biofilm regulation [35]. However, *rpoE* and *rybB* were not among the identified NsrR-bound promoters, suggesting that their repression may occur via an indirect mechanism [35].

To clarify how NsrR represses *rpoE* and *rybB*, we considered three potential mechanisms: (1) indirect repression of *rybB* via reduced *rpoE* expression, (2) inhibition of RpoE activity through anti-sigma-like interactions, and (3) direct repression of both genes by a mechanism distinct from NsrR’s NO-regulated regulon. Herein, we show that NsrR represses RpoE-dependent transcription, with kinetics and strength comparable to those of RseA. NsrR overexpression blocks RpoE autoregulation and downstream target induction, even when RpoE is expressed from a plasmid. Moreover, NsrR overexpression phenocopies Δ*rpoE*, producing filamentous cell morphology and impaired growth. We further demonstrate that NsrR physically interacts with RpoE in bacterial two-hybrid assays, and AlphaFold3 modeling predicts a plausible interaction interface [36]. To dissect these mechanistic models, we employed a combination of genetics, transcriptional reporters, two-hybrid interaction assays, and structural prediction to test whether NsrR modulates σ^E^ activity through direct interaction, indirect repression, or both. Together, these findings support a model in which NsrR acts as a noncanonical modulator of RpoE, functioning outside its traditional NO-responsive role to shape the envelope stress response.

## 2. Results

### 2.1. NsrR Overexpression Represses σ^E^-Dependent Promoters and Disrupts Cell Division

**Figure 1:**
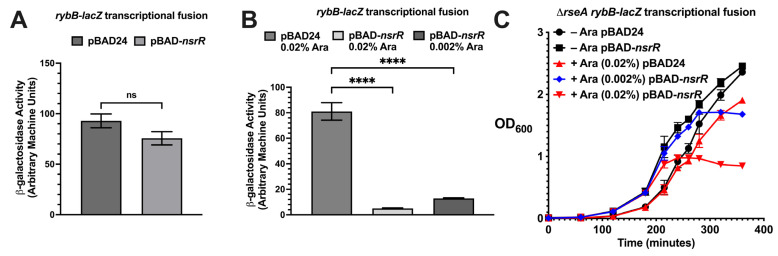
The effect of NsrR overexpression on *rybB* expression and bacterial growth. (**A**) β-galactosidase activity of strain KMT12078 (Δ*rseA rybB*-*lacZ*) harboring either empty vector (pBAD24, KMT12090) or pBAD24-*nsrR* (KMT12091), grown in LB + ampicillin (50 μg/mL) with either 0.002% or 0.02% arabinose. Cultures were harvested at OD_600_ 1.6–2.3. (**B**) Dose-dependent repression of *rybB*-*lacZ* by NsrR overexpression. KMT12078 carrying pBAD24 or pBAD24-*nsrR* was grown in LB + ampicillin with 0.002% or 0.02% arabinose. (**C**) Growth curves of KMT12078 harboring pBAD24 or pBAD24-*nsrR* in LB + ampicillin with indicated arabinose concentrations. OD_600_ was monitored over time. Significance: **** *p* < 0.0001, ns = not significant.

**Figure 2:**
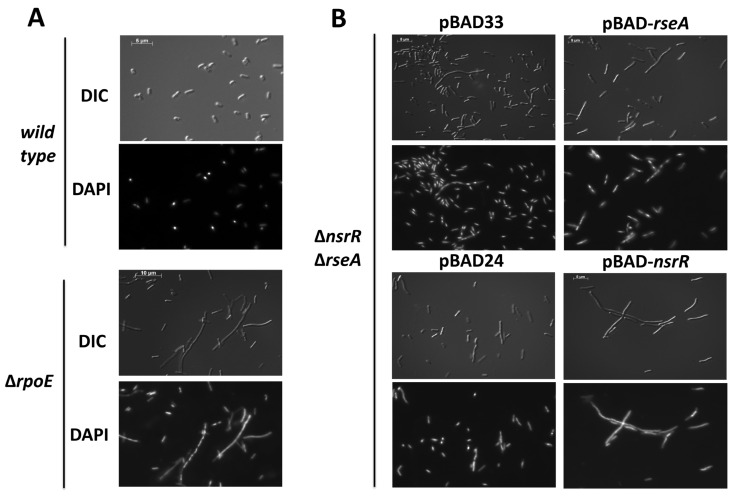
The cellular morphology effects of NsrR overexpression and RpoE inactivation. Differential interference contrast (DIC) and DAPI-stained fluorescence microscopy images of (**A**) wild-type (KMT414) and Δ*rpoE* (KMT471) cells grown to OD_600_ ~1.0; (**B**) Δ*nsrR* Δ*rseA* (KMT473) cells transformed with vector control—pBAD33 or pBAD24, pBAD33-*rseA*, or pBAD24-*nsrR*, grown in LB + appropriate antibiotics and 0.02% arabinose to OD_600_ 0.8–1.0 before imaging.

As previously reported, overproduction of NsrR was sufficient to repress *rybB*-*lacZ* and *rpoE270*_P2_-*lacZ* fusion in the absence of *rseA* [15]. NsrR was expressed from an arabinose-inducible promoter, and the addition of arabinose was necessary to see repression (Figure 1A,B; 1A without arabinose and 1B with arabinose). The overexpression of *nsrR* in the presence of 0.02% arabinose resulted in a severe growth defect (Figure 1C); the overexpression of *nsrR* in the presence of 0.002% arabinose did not inhibit growth as rapidly (Figure 1C). These growth defects prompted us to hypothesize that NsrR impairs cell division or chromosome segregation. A previous report demonstrated aberrant cellular morphology phenotypes upon inhibition of σ^E^ activity because of RseA overexpression [37]. We hypothesized that we would observe aberrant cellular morphology because of NsrR overexpression, due to the NsrR repression of *rpoE*. Therefore, we examined the cellular morphology upon the overexpression of NsrR, using RseA overexpression and the absence of *rpoE* as positive controls. For our positive control, wild-type and ∆*rpoE* cells were grown to the mid-exponential phase (OD_600_ = 0.5), and aliquots of the cultures were then harvested and analyzed by differential interference contrast and fluorescence microscopy using DAPI staining. The absence of *rpoE* led to severe cellular filamentation in a significant proportion of the population of cells (Figure 2A). For the analysis of the NsrR overexpression, we transformed a ∆*nsrR* ∆*rseA* double mutant with a plasmid containing an arabinose-inducible allele of *rseA* or *nsrR*. We then grew the cells in LB supplemented with ampicillin or chloramphenicol and 0.002% arabinose to an OD_600_ of 0.7–1.0. We then harvested aliquots of these cultures and analyzed them by differential interference contrast and fluorescence microscopy using DAPI staining (Figure 2B). Consistent with previous reports [37], overexpression of RseA leads to the formation of membrane blebs (Figure 2B). In addition, there was also some cellular filamentation in a proportion of the cells. The overexpression of *nsrR* led to a severe cellular filamentation that was seen in many of the cells (Figure 2B). Taken together, this suggests that *nsrR* overexpression results in cellular morphology defects that are very similar to those seen upon removing or inactivating *rpoE*.

### 2.2. NsrR and RseA Independently Repress rybB and rpoE, Supporting a Parallel Regulatory Model

**Figure 3:**
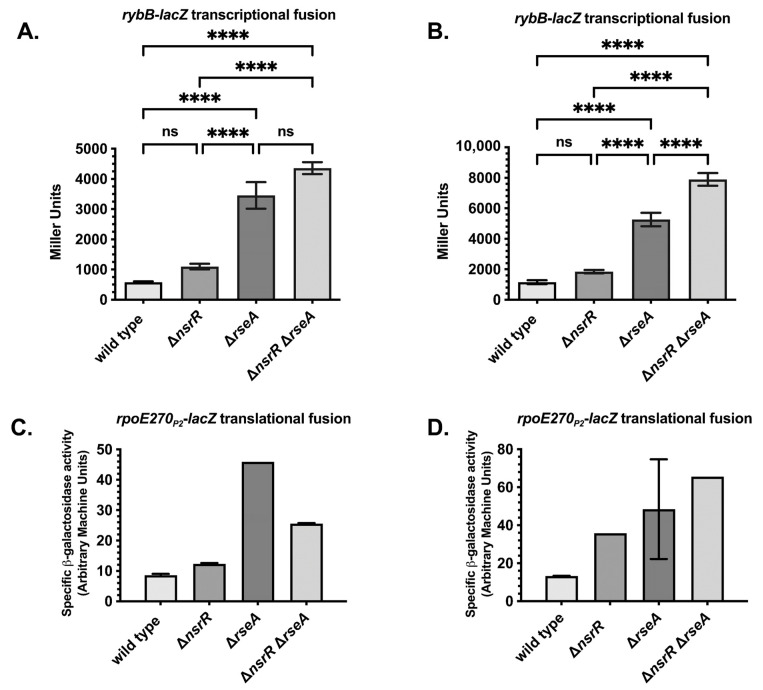
The expression of *rybB* and *rpoE* in Δ*nsrR*, Δ*rseA*, and double mutants. (**A**,**B**) β-galactosidase activity of *rybB-lacZ* transcriptional fusion strains in exponential (**A**) and stationary (**B**) phases. Strains include wild-type (KMT12093), Δ*rseA* (KMT12094), Δ*nsrR* (KMT12099), and Δ*nsrR ΔrseA* (KMT12100) grown in LB at 37 °C. (**C**,**D**) β-galactosidase activity of *rpoE270P2-lacZ* translational fusion strains with the same genotypes (KMT14000, KMT14001, KMT14021, and KMT14022) and growth conditions, measured during exponential (**C**) and stationary (**D**) phases of growth. Bars represent the means of at least three independent biological replicates. Statistical analysis was performed using one-way ANOVA (GraphPad Prism 10). Significance: **** *p* < 0.0001, ns = not significant.

To assess the physiological significance of the effects of *nsrR* on *rybB* and *rpoE*, we tested the effect of Δ*nsrR* on the expression of *rybB* and *rpoE*. We transduced Δn*srR*::*tet* mutations into strains carrying *rybB*-*lacZ* transcriptional fusions and *rpoE270*_P2_-*lacZ* translational fusions, in wild-type or Δ*rseA* genetic backgrounds, and measured their activity at various stages of growth. As expected, Δ*rseA* significantly increased the expression levels of both fusions, both in the exponential (Figure 3A,C) and stationary phases of growth (Figure 3B,D). The absence of *nsrR* resulted in an approximate 2-fold increase in *rybB*-*lacZ* expression in *rseA*^+^ cells, which was statistically significant, in both the exponential and stationary phases of growth (Figure 3A,B). The statistically significant increase in the expression of *rybB*-*lacZ* in *rseA* mutants is much greater at approximately 7-fold in the exponential phase and 4-fold in the stationary phase (Figure 3A,B). In cells deleted for *rseA*, Δ*nsrR* further increased the activity of *rybB*-*lacZ* (Figure 3A,B), confirming and extending our previous finding that NsrR repression is independent of RseA. However, this increase was not statistically significant in exponential-phase cultures. The Δ*nsrR* mutant strain of *rpoE270*_P2_-*lacZ* also had a statistically significant increase in expression versus the wild-type strain (Figure 3C,D). While this increase was less than 2-fold in the exponential phase, there was a 2–3-fold increase in the stationary phase (Figure 3C,D). In contrast with the *rybB*-*lacZ* fusion strain, the Δ*nsrR* Δ*rseA* double mutant did not exhibit an increase in activity in comparison to the Δ*rseA* mutant in exponential phase for reasons that are not clear. In stationary phase, there is a slight additive increase that is not likely to be statistically significant (Figure 3C,D). Overall, these results confirm that the multicopy repression previously seen reflects a physiologically relevant role of NsrR in the regulation of the σ^E^ response.

### 2.3. NsrR Interferes with RpoE-Dependent Promoter Activation in an Inducible Expression System

**Figure 4:**
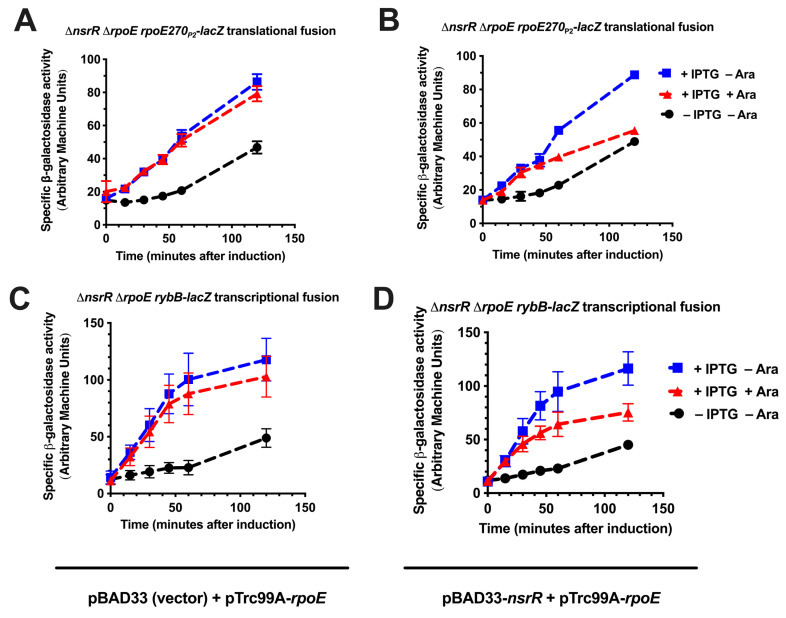
Inducible expression of RpoE and repression by NsrR in fusion reporter assays. (**A**,**B**) Time-course analysis of *rpoE270P2-lacZ* translational fusion activity in strain KMT14022 (Δ*nsrR ΔrseA*). Cells contained either pBAD33 (vector control, (**A**)) (KMT14032) or pBAD33-*nsrR* (KMT14033) (**B**) along with pCL245 (IPTG-inducible *rpoE* on pTrc99A). Cultures were grown to OD_600_ = 0.5 and then induced with 100 µM IPTG or with IPTG plus 0.2% arabinose. β-galactosidase activity was measured every 15 min for 120 min post induction. (**C**,**D**) Time-course analysis of *rybB-lacZ* transcriptional fusion activity in strain KMT12131 (Δ*nsrR ΔrseA*) treated in the same manner as described above. Cultures contained either pBAD33 (a vector control), (**C**) (KMT12133), or pBAD33-*nsrR* (KMT12134) (**D**), along with pCL245. X/Y data points represent the means of at least three independent biological replicates. Error bars represent SEMs of at least three independent biological replicates.

To test this hypothesis, we measured the ability of NsrR to repress *rpoE* or *rybB* promoters in strains in which the sole source of *rpoE* and *nsrR* was from compatible plasmids with different inducible promoters (on the pTrc99A-*rpoE* and pBAD33-*nsrR* plasmids, respectively). To execute the experiment, we grew the cultures to an OD_600_ of 0.5 and then added IPTG or IPTG and arabinose and isolated samples every 15 min after induction for 120 min (Figure 4). For both the *rpoE270*_P2_-*lacZ* translational and *rybB*-*lacZ* transcriptional fusions, the fusions were active in the absence of IPTG and increased over time, suggesting that there is leaky induction of *rpoE* from the plasmid-borne IPTG-inducible allele of *rpoE* (Figure 4A–D, black line). Upon the addition of IPTG, the activities of both the *rpoE270*_P2_-*lacZ* translational and *rybB*-*lacZ* transcriptional fusions increased over time and were significantly greater than the activities in the absence of IPTG, confirming the σ^E^-dependent activation of *rpoE270*_P2_-*lacZ* and *rybB*-*lacZ*, as expected (Figure 4A–D, blue line). This induction was unaffected by the induction of the pBAD33 vector with arabinose (Figure 4A,C, red line). However, the induction of NsrR decreased the activities of both the *rpoE270*_P2_-*lacZ* and *rybB*-*lacZ* fusions, in comparison to the activities of the fusions in the presence of IPTG alone (compare red to blue lines in Figure 4B,D). Therefore, overexpression of NsrR can inhibit the σ^E^ induction of *rybB* and *rpoE*_P2_ promoters and is not simply acting at the level of RpoE synthesis. This would presumably occur through the direct interaction of NsrR with either the σ^E^-dependent *rybB* and *rpoE*_P2_ promoters through interaction with the σ^E^ protein itself in a manner analogous to RseA.

### 2.4. NsrR Represses Additional σ^E^-Regulated sRNAs

**Figure 5:**
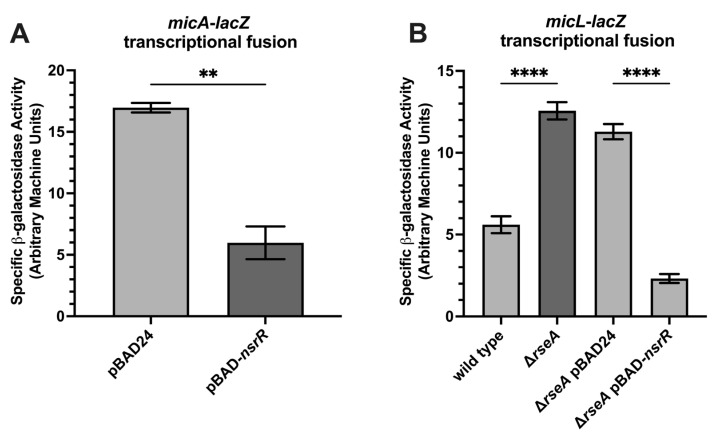
Reporter activity from *micA* and *micL* fusions in strains overexpressing NsrR. (**A**) β-galactosidase activity from a *micA-lacZ* transcriptional fusion strain containing either a vector control (pBAD24–KMT12294) or *nsrR* expressed from an arabinose-inducible plasmid (pBAD24-*nsrR*: KMT12295). Cultures were grown with or without 0.02% arabinose and harvested at the late exponential phase of growth. Statistical analysis was performed using an unpaired *t*-test. Significance: ** *p* < 0.0010. (**B**) β-galactosidase activity from a *micL-lacZ* transcriptional fusion strain in a wild type (JN100) Δ*rseA* mutant background (JN101) with an empty vector control-pBAD24 (JN102) or an *nsrR* overexpression plasmid (JN103), grown and harvested as in (**A**). Statistical analysis was performed using one-way ANOVA (GraphPad Prism 10). Significance: **** *p* < 0.0001.

To determine whether NsrR represses additional σ^E^-regulated small RNAs beyond *rybB*, we examined *micA* and *micL* expression using *lacZ* transcriptional reporter fusions. The *micA-lacZ* strain harboring either pBAD24 or pBAD24-*nsrR* was grown to the late exponential phase of growth in LB supplemented with 0.02% arabinose. Overexpression of *nsrR* significantly reduced the *micA-lacZ* activity (Figure 5A). Similarly, *nsrR* overexpression repressed the *micL-lacZ* expression in a Δ*rseA* background (Figure 5B), suggesting that this repression occurs independently of RseA-mediated sequestration. These findings extend NsrR’s regulatory reach within the σ^E^ regulon and raise the possibility that, like RseA, NsrR may function in a parallel pathway to modulate σ^E^ activity. This observation prompted us to compare repression kinetics of NsrR and RseA on the *rybB* promoter.

### 2.5. NsrR Does Not Exhibit Robust Binding to σ^E^ Target Promoters In Vitro

To test whether NsrR directly represses *rpoE* or *rybB* through promoter binding, we performed electrophoretic mobility shift assays (EMSAs), DNase I footprinting, and in vitro transcription assays using purified NsrR and radiolabeled promoter fragments. However, none of these approaches revealed consistent or robust DNA-binding activity under standard conditions. These negative results suggest that NsrR may not repress these promoters via classical DNA binding or that such interactions are weak, transient, or context dependent—potentially requiring stress signals or co-factors absent in vitro. These findings are consistent with previous ChIP-seq studies that did not identify *rpoE* or *rybB* as direct NsrR targets [38]. To address this possibility, we plan to apply recently developed chromatin-profiling methods with significantly enhanced sensitivity over the previous technical standard (ChIP-seq), under defined stress conditions, as described in the discussion, to evaluate the potential for condition-dependent promoter occupancy.

### 2.6. NsrR Phenocopies RseA Repression Kinetics at the rybB Promoter

**Figure 6:**
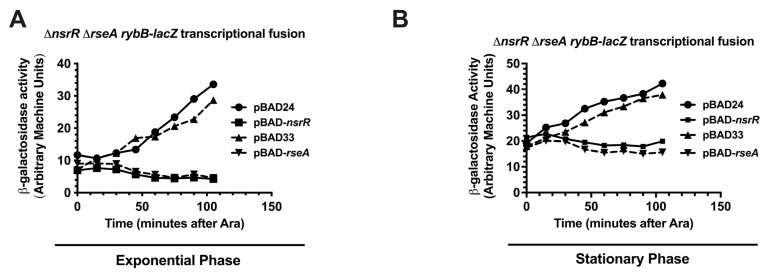
Time-course repression of *rybB* by RseA and NsrR. Strain KMT12100 (Δ*rseA* Δ*nsrR rybB-lacZ*) was transformed with arabinose-inducible plasmids expressing either *nsrR* (pBAD24-*nsrR*: KMT12122) or *rseA* (pBAD33-*rseA*: KMT12124), or with their respective vector controls (pBAD24 and pBAD33: KMT12121 and KMT12123, respectively). Overnight cultures were diluted in LB supplemented with either 0.2% glucose and ampicillin (for pBAD24 constructs) or chloramphenicol (for pBAD33 constructs) to repress expression from the arabinose inducible promoter. Cultures were grown to OD_600_ = 0.5 (**A**) the exponential phase of growth) or to OD_600_ = 1.0 (**B**) the stationary phase of growth), harvested by filtration, and resuspended in pre-warmed LB containing 0.02% arabinose to induce expression of *nsrR* or *rseA*. Aliquots were collected at 0 (pre-induction), 5, 10, 15, 20, 25, 30, and 45 min post induction for the measurement of *rybB-lacZ* activity. Statistical analysis was performed using one-way ANOVA (GraphPad Prism 10).

To test the relative repression kinetics of RseA and NsrR on *rybB* transcription (as a representative of the σ^E^ regulon with one of the strongest promoters), we activated the *rybB*-*lacZ* transcriptional fusion followed by the rapid induction of NsrR and RseA—measuring β-galactosidase activity every 15 min for 105 min. The rationale behind this experiment is that if NsrR directly represses σ^E^-dependent promoters, the kinetics of NsrR repression of *rybB* will be similar or identical to RseA repression. If NsrR acts indirectly (represses or activates the synthesis of another regulator, for instance), we would expect NsrR repression to be significantly slower than repression by the RseA anti-sigma factor, reflecting the time to either deplete or accumulate the direct regulator. To tightly control *nsrR* and *rseA* levels for this assay, we used a *rybB*-*lacZ* transcriptional fusion strain with a Δ*nsrR* Δ*rseA* genetic background transformed with vectors or with plasmids carrying arabinose-inducible alleles of *nsrR* or *rseA*. We then grew these strains and the vector controls in LB supplemented with 0.2% glucose to an OD_600_ of 0.5 (exponential—Figure 6A) or 1.0 (stationary—Figure 5B) and shifted the cells to LB supplemented with 0.2% arabinose. We analyzed culture aliquots for specific β-galactosidase activity at 15 min intervals, following arabinose induction, up to 120 min. For the cultures shifted in exponential phase, the specific β-galactosidase activity of the *rybB*-*lacZ* transcriptional fusion strain containing the pBAD24 or pBAD33 vector controls increased over time, consistent with the activity of σ^E^-dependent promoters. The activity in the strain containing pBAD33-*rseA* did not increase, and slightly decreased, consistent with the known direct negative regulation of σ^E^ activity by RseA. The strain containing pBAD24-*nsrR* was very similar in behavior to the RseA-expressing strain. These kinetics suggest that NsrR directly regulates σ^E^-dependent transcription. Taken together, these observations prompted us to investigate a potential NsrR-RpoE protein–protein interaction mechanism.

### 2.7. NsrR–RpoE Interaction Confirmed by Bacterial Adenylate Cyclase Two-Hybrid (BACTH) and Explored via AlphaFold3 Multimer Modeling

**Figure 7:**
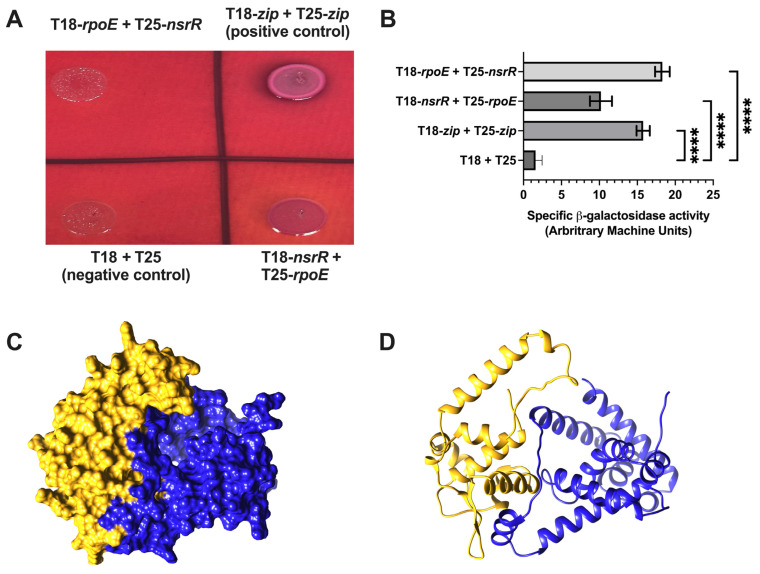
Analysis of the NsrR–RpoE interaction using The Bacterial Adenylate Cyclase Two-Hybrid assay and AlphaFold3 structural modeling. (**A**) Bacterial adenylate cyclase two-hybrid (BACTH) assay showing colony color on MacConkey Maltose agar. NsrR and RpoE were fused to the T18 and T25 fragments of adenylate cyclase in both orientations. Plates include vector-only negative control and leucine zipper positive control. (**B**) Quantification of β-galactosidase activity from the BACTH assay. Constructs expressing T18–NsrR + T25–RpoE and the reciprocal T18–RpoE + T25–NsrR were compared to controls. Bars represent the means of at least three biological replicates; error bars indicate SEM. Statistical analysis was performed using one-way ANOVA (GraphPad Prism 10). Significance: **** *p* < 0.0001. (**C**) The ribbon diagram of the NsrR homodimer model. (**D**) The best AlphaFold3 model of the NsrR dimer and RpoE with pTM 0.53 and ipTM 0.39. NsrR dimer is in purple and coral and has the same orientation as in (**C**). RpoE is in rainbow coloring, where the N terminal is in blue and the C terminal is in red.

Given the rapid and parallel repression kinetics of NsrR and RseA at the *rybB* promoter, we hypothesized that NsrR may regulate σ^E^ activity post-translationally through a direct interaction with RpoE, analogous to the canonical anti-sigma factor (RseA). We employed the BACTH system to test for interactions and used AlphaFold3 multimer modeling to visualize potential interaction surfaces. The BACTH system detects protein–protein interactions based on cAMP-dependent transcriptional activation. Reciprocal fusions of NsrR and RpoE to the T18 and T25 adenylate cyclase fragments produced detectable colony color on MacConkey maltose agar comparable to that of the leucine zipper positive control (Figure 7A). Quantitative β-galactosidase assays confirmed a significant interaction between NsrR and RpoE relative to vector-only controls (Figure 7B). To investigate the molecular nature of this interaction, we used AlphaFold3 multimer modeling, which offers improved capabilities for predicting multi-subunit complexes and flexible protein interfaces, to model the NsrR–RpoE interaction [36]. Direct modeling of the NsrR–RpoE heterodimer yielded low confidence scores (pTM 0.48, ipTM 0.17; Figure S1A), suggesting model uncertainty. However, consistent with prior reports that NsrR functions as a dimer [39], AlphaFold3 confidently predicted the NsrR homodimer (pTM 0.83, ipTM 0.82; Figure 7C). We then modeled the interaction between the NsrR dimer and RpoE. Among five predicted models (Figure 7D and Supplemental Figure S1B–E), the NsrR dimer structure was stable, but the RpoE orientation varied, likely due to the presence of a flexible linker region (residues 92–116). When RpoE is a part of the RNA polymerase holoenzyme (e.g., in complex with RpoA, RpoB, RpoC, and RpoZ; PDB: 6JBQ) [40], this linker is extended, allowing its σ^70^ domains to contact promoter DNA. In contrast, when RpoE is bound to RseA (PDB: 1OR7—Supplemental Figure S1G) [40], the linker adopts a helical structure, bringing its N- and C-terminal domains into close proximity. These differences highlight the challenge of predicting flexible domain orientations with current algorithms. While AlphaFold3 offers valuable structural insight, experimental validation through mutagenesis or high-resolution structural methods will be necessary to confirm the specific mode of the NsrR–RpoE interaction.

## 3. Discussion

### 3.1. NsrR Is a Dual Regulator of Envelope and Nitrosative Stress Responses

Our study reveals an expanded role for the nitric oxide (NO)-sensing transcriptional regulator (NsrR) in *Escherichia coli*, extending beyond its canonical repression of NO detoxification and iron–sulfur cluster maintenance genes. We demonstrate that NsrR modulates the σ^E^ (RpoE) envelope stress response by repressing σ^E^-regulated targets, including *rybB*, *micA*, *micL*, and *rpoE* itself. These effects occur independently of RseA, the membrane-bound anti-sigma factor that typically sequesters σ^E^ under non-stress conditions, suggesting that NsrR functions through a parallel regulatory mechanism.

Rather than constraining σ^E^ under basal conditions—when it is already sequestered—NsrR may act after the initial activation of σ^E^, functioning as a secondary brake that dampens prolonged or excessive envelope stress signaling. This dual role positions NsrR as a redox-sensitive checkpoint that coordinates nitrosative and envelope stress responses. By modulating σ^E^ output following activation, NsrR helps to maintain the balance between outer membrane protein (OMP) synthesis and envelope remodeling during cell elongation and division—an essential equilibrium for bacterial viability.

### 3.2. RybB Repression as a Model for the NsrR-Dependent Modulation of σ^E^

We used the small RNA *rybB*, one of the most prominent σ^E^ targets, as a model for dissecting the NsrR-dependent modulation of the envelope stress response. NsrR overexpression strongly repressed *rybB*-*lacZ* activity and phenocopied σ^E^ inactivation, producing filamentous morphology and growth defects. Conversely, Δ*nsrR* resulted in elevated expression of both *rybB* and *rpoE*, with additive effects in a Δ*rseA* background. This suggests that NsrR and RseA exert independent yet convergent repression of σ^E^ activity.

Importantly, NsrR also repressed *micA* and *micL*, two additional σ^E^-regulated sRNAs, highlighting a broader role in the post-transcriptional branch of the σ^E^ regulon. These sRNAs fine-tune OMP levels by promoting the degradation of outer membrane protein mRNAs. Thus, NsrR’s repression of these regulatory RNAs suggests it serves not only as a transcriptional repressor but also as a master regulator that constrains downstream RNA-based stress adaptations to limit energy expenditure and prevent deleterious remodeling of the cell envelope.

### 3.3. NsrR May Act as a Noncanonical Anti-σ^E^ Factor

Given the kinetic and phenotypic similarities between NsrR and RseA repression of σ^E^-dependent promoter activity, we hypothesized that NsrR could directly modulate σ^E^ activity via protein–protein interactions. Supporting this model, bacterial two-hybrid assays revealed a direct interaction between NsrR and RpoE, further corroborated by multimer modeling (Figure 6), which predicted a plausible NsrR–RpoE interface. These data support a model in which NsrR may act as a noncanonical anti-σ^E^ factor, directly antagonizing the σ^E^ function through physical sequestration or inhibition.

Such dual-function regulators—combining transcriptional repression with direct sigma factor binding—are rare but increasingly recognized in bacterial stress networks. This model draws conceptual parallels to the *Bacillus subtilis* SigB system, where a complex cascade of sigma, anti-sigma, and anti-anti-sigma factors modulates stress responses [41,42]. In *E. coli*, a redox-sensitive interaction between NsrR and σ^E^ could serve as a novel regulatory checkpoint, integrating environmental NO signals with envelope stress signaling.

### 3.4. Physiological and Evolutionary Implications

The ability of NsrR to repress σ^E^ targets may reflect an adaptive mechanism that prioritizes NO detoxification and redox homeostasis over envelope repair during transient stress. In host environments, such as macrophages—where NO exposure and membrane perturbation often co-occur—this regulatory logic ensures that envelope stress responses are only activated when both redox and envelope stress thresholds are surpassed. Under non-lethal conditions, NsrR-mediated repression may help to avoid the futile or energetically costly induction of envelope repair pathways.

Upon the oxidation of NsrR’s [Fe–S] cluster, de-repression of the σ^E^ regulon would allow the appropriate induction of protective and reparative functions. This model supports a broader framework in which NsrR functions not only as a sensor of NO but also as a temporal gatekeeper for multi-stress adaptation, fine-tuning bacterial survival strategies under compound environmental challenges.

### 3.5. Open Questions and Future Directions

These findings suggest that NsrR may regulate σ^E^ through both direct protein–protein interactions and indirect regulatory effects, extending its role beyond the nitric oxide response. While the precise mechanisms remain to be defined, our data support a model in which NsrR functions as a noncanonical anti-sigma factor. Future studies will be required to clarify the molecular interface, regulatory scope, and physiological triggers of this interaction.

Notably, it remains unclear whether envelope stress directly modulates NsrR expression or activity. Although NsrR is classically regulated by nitric oxide via the oxidation of its [Fe–S] cluster, envelope stress or altered σ^E^ activity may influence *nsrR* stability or activity. Supporting this, Nicoloff et al. (2017) [43] identified a point mutation in *nsrR*, in a screen for suppressors of high σ^E^ activity in a Δ*rseA* genetic background. While the functional consequence of this point mutation is unknown, it is possible that it could augment repression of σ^E^ synthesis or activity. Such a mechanism would position NsrR as both a redox-responsible regulator and a feedback inhibitor that retrains prolonged σ^E^ activity under envelope stress conditions.

Given the conservation of both NsrR and σ^E^ across Gram-negative bacteria, analogous cross-regulatory mechanisms may operate in pathogens such as *Salmonella*, *Klebsiella*, and *Pseudomonas*. Dissecting how NO-sensing regulators influence envelope integrity pathways could uncover novel strategies to disrupt bacterial stress tolerance, persistence, and virulence.

## 4. Materials and Methods

### 4.1. Media and Growth Conditions

*E. coli* strains were cultured in LB (Lennox, Richardson, TX, USA) medium or on LB agar plates with appropriate antibiotics and inducers as described. For plasmid selection and maintenance, antibiotics were added at the following final concentrations: kanamycin (25 μg/mL), chloramphenicol (25 μg/mL), tetracycline (25 μg/mL), and ampicillin (100 μg/mL). P*_BAD_*-regulated promoters were repressed by supplementing media with 0.2% glucose and induced with L-arabinose at final concentrations of 0.02% or 0.002%. IPTG was used at 10 or 100 μM for the induction of plasmid-encoded *rpoE*. Plasmids were introduced via TSS transformation or electroporation. P1*vir* phage transduction was used to transfer chromosomal mutations following standard protocols [44].

### 4.2. Strains, Plasmids, and Oligonucleotides

All the strains were derived from *E. coli* K-12 MG1655. Full lists of strains, plasmids, and primers are provided in Table 1, Table 2 and Table 3.

#### 4.2.1. Chromosomal Deletion–Insertion Mutations of *nsrR* and *rpoE*

A Δ*nsrR*::*tet* mutation was generated by λ-Red recombineering [44,46,47,48]. A tetracycline resistance cassette flanked by 50 bp homology arms upstream and downstream of *nsrR* was amplified using primers KT237 and KT238 to create a linear allelic exchange substrate (AES) for recombination. The PCR product was electroporated into strain NM200 (mini-*λ*::*cat*) after the induction of λ-Red recombinase at 43.5 °C for 15 min [47]. Transformants (putative recombinants) were selected on LB–tetracycline plates and confirmed by PCR and sequencing. A Δ*rpoE*::*kan* mutation was generating in an identical manner with minor changes. Briefly, the AES was amplified using primers KT926 and KT927. And transformants (putative recombinants) were selected on LB-kanamycin plates.

#### 4.2.2. Construction of Plasmid-Based Arabinose-Inducible *nsrR* and *rseA* Alleles

The *nsrR* gene was amplified from MG1655 genomic DNA, using primers KT191 and KT192, and digested with *Eco* RI and *Pst* I before ligation into pBAD24. Primers KT906 and KT907 were used for the PCR amplification of *nsrR,* which was digested with *Sac* I and *Pst* I before ligation into pBAD33. The *rseA* gene was amplified with primers KT95 and KT75 and digested with *Sac* I and *Xba* I and cloned similarly into pBAD33. The constructs were verified by restriction digestion and sequencing. The primers are listed in Table 3.

#### 4.2.3. Construction of Bacterial Adenylate Cyclase Two-Hybrid Assay (BACTH) Plasmids

The *nsrR* and *rpoE* open reading frames were PCR-amplified using the gene-specific primers listed in Table 3 and cloned in-frame with either the T18 or T25 domain of adenylate cyclase. Cloning was performed using HiFi Assembly Master Mix (New England Biolabs, Ipswich, MA, USA) according to the manufacturer’s instructions. The pEB355 and pEB354 vectors were used to generate T18 and T25 fusion constructs, respectively. Specifically, T18-*nsrR* was constructed using primers KT2223 and KT2224, and T18-*rpoE* was constructed using KT2225 and KT2226. T25-*nsrR* and T25-*rpoE* were constructed with primer pairs KT2227/KT2228 and KT2229/KT2230, respectively. All the plasmid constructs were confirmed by restriction digestion and Sanger sequencing and were subsequently used to assess NsrR–RpoE protein–protein interactions in the BACTH system.

### 4.3. Bacterial Two-Hybrid (BACTH) Assay

Protein–protein interactions were assessed using the bacterial adenylate cyclase two-hybrid (BACTH) system [49,50]. Plasmid constructs encoding in-frame fusions of *nsrR* or *rpoE* to the T18 or T25 domains of adenylate cyclase were introduced to *E. coli* BTH101 by electroporation. The following plasmid pairs were tested: pEB355 and pEB354 (empty vector (negative control)), pEB355-*zip* and pEB354-*zip* (positive control), pEB355-*nsrR* + pEB354-*rpoE*, and pEB355-*rpoE* + pEB354-*nsrR*. These constructs generated reciprocal T18 and T25 fusions of the two proteins of interest. Electrocompetent BTH101 cells were prepared by washing log-phase cultures three times with ice-cold sterile water. Plasmid mixtures were electroporated using the Bio-Rad MicroPulser™, Bio Rad, Hercules, CA, USA (Ec1 “Bacteria” setting), and cells were recovered in LB for 1 h at 30 °C. Aliquots were plated on LB agar containing ampicillin and kanamycin for the selection of double transformants. Selected colonies were grown overnight in liquid LB supplemented with the same antibiotics and 100 μM IPTG to induce the expression of the fusion proteins. Cultures were spotted onto MacConkey maltose agar plates and incubated at 30 °C for 24 to 48 h. The colony color was used as a qualitative measure of interaction-dependent cAMP restoration and lac operon activation. Quantitative β-galactosidase assays were performed, as described below, on parallel cultures to confirm and measure the interaction strength.

### 4.4. Quantitative β-Galactosidase Assays

β-galactosidase assays were performed using a standard protocol [51] or in 96-well plates, as previously described [52]. For the standard protocol, the specific β-galactosidase activity was expressed as Miller units, as previously described [51]. Cultures were harvested at the exponential or stationary phase, permeabilized, and incubated with ONPG. Absorbance at 420 nm measured using a SpectraMax 250 plate reader (Marshall Scientific, Hampton, NH, USA). The specific β-galactosidase activity was expressed as arbitrary machine units and defined as the slope of OD_420_/OD_600_. The results represent the means of at least three biological replicates.

### 4.5. Microscopy Experiments

Cells were grown to the mid-log phase of growth, fixed, and stained with DAPI. The morphology was analyzed using differential interference contrast (DIC) and fluorescence microscopy. Images were acquired on a Axio Imager.M2 upright fluorescence microscope (Carl Zeiss Microscopy, LLC, White Plains, NY, USA), using 100× magnification, and processed using Zeiss Zen software version 3.12 (Carl Zeiss Microscopy, LLC, White Plains, NY, USA).

### 4.6. Protein–DNA Interaction Assays

Electrophoretic Mobility Shift Assays (EMSAs), DNase I footprinting, and in vitro transcription assays were performed using purified NsrR, as well as RpoE reconstituted with purified RNA polymerase holoenzyme and radiolabeled promoter fragments from *rpoE* and *rybB*. Assays were performed under standard binding conditions. No robust or specific binding was observed under any condition tested. The protocol details are available upon request.

### 4.7. Structural Modeling Using AlphaFold3

Protein–protein interaction modeling between NsrR and RpoE was performed using AlphaFold3. Amino acid sequences of full-length *E. coli* NsrR and RpoE were used as input to the AlphaFold server with default options. Top5 models and experimental structures 6JBQ and 1OR7 in PDB were download and examined in UCSF ChimeraX [53] to generate the illustrations.

### 4.8. Statistical Analysis

All the statistical analyses were performed using GraphPad Prism 10. One-way ANOVA was used to assess group differences. Bartlett’s or Brown–Forsythe tests were applied to evaluate assumptions of equal variance, depending on the dataset. When comparing two groups, unpaired *t*-tests were used. Unless otherwise noted, the data represent the mean of at least three independent biological replicates. Bars and connected scatter points display group means; error bars represent the standard error of the mean (SEM). Specific statistical tests and significance thresholds are noted in individual figure legends.

## Figures and Tables

**Table 1:** NsrR targets coregulated by RpoE or CpxR or related to envelope homeostasis.

σ^E^ Regulon	CpxR Regulon	Envelope Homeostasis
*rpoE* ^1^	*spy* ^2^	*mipA* (*yeaF*) ^2,6^
*rybB* ^1^	*dsbA* ^2^	*yfhB* (*pgpC*) ^2^
*mica* ^1^		*mepH* (*ydhO*) ^2^
*htrG* (*ygiM/ecfG*) ^2,3,4^		*ygiH* (inner membrane protein) ^2^
*hcp* ^2,3,5^		*cbrB* ^2^
*smpA* ^2,3^		

1—This work; 2—[38]; 3—RpoE regulon [12]; 4—Regulated by MicA [16]; 5—*hcp* is a major NsrR target [38]; 6—MipA acts as a multicopy suppressor of mutations that decrease rybB promoter activity [15].

**Table 2:** Bacterial Strains.

Strain or Plasmid	Genotype	Source or Reference
Strains		
MG1655	*Esherichia coli* K12	Thomspon et al. 2007 [15]
DJ480	MG1655 Δ*lacIZYA*	Thomspon et al. 2007 [15]
JN100	NM580 *micL-lacZ* WT	NM580 x *micL-lacZ* PCR gBlock by Electroporation
JN101	NM580 *micL-lacZ* Δ*rseA*::*kan*	JN100 x P1 (Δ*rseA*::*kan*)
JN102	NM580 *micL-lacZ* Δ*rseA*::*kan* pBAD24	JN101 + pBAD24 (TSS Transformation)
JN103	NM580 *micL-lacZ* Δ*rseA*::*kan* pBAD-*nsrR*	JN102 +pBAD-*nsrR* (TSS Transformation)
KMT243	DJ480 mini-l::*tet* Δ*rseA*::*kan*	KMT295 (induced) + Δ*rseA*::*kan* (PCR w/oligos KT76 and KT77) cassette via electroporation
KMT414	MG1655 Δ*lacZYAfrtfrt lacI*^Q^ Δ*ara714* ΔP*araE*::*frtfrt*P_CP18_*araE*	NM534 obtained from Nadim Majdalani as a recipient for *frtkanfrt*-PLlacO-rpoE
KMT422	DJ480 Δ*nsrR*::*tet*	This work, x Δ*nsrR*::*tet* PCR
KMT456	*E. coli* BTH101 pEB354 (T25 linker) Kan^R^	T25 linker vector, *EcoR* I/*Xho* I, from Aureilia Battesti, Ph.D.
KMT457	*E. coli* BTH101 pEB354 (T25-Zip) Kan^R^	Positive control for pEB354 (T25 linker) Aureilia Battesti, Ph.D.
KMT458	*E. coli* BTH101 pEB355 (T18 linker) Amp^R^	T18 linker vector, *EcoR* I/*Xho* I, from Aureilia Battesti, Ph.D.
KMT459	*E. coli* BTH101 pEB355 (T18-Zip) Amp^R^	Positive control for pEB355 (T18 linker) Aureilia Battesti, Ph.D.
KMT470	MG1655 Δ*lacZYAfrtfrt lacI*^Q^ Δ*ara714* ΔP*araE::frtfrt*P_CP18_*araE* Δ*nsrR*::*tet*	KMT414 x P1 (Δ*nsrR*::*tet*)
KMT471	MG1655 Δ*lacZYAfrtfrt lacI*^Q^ Δ*ara714* ΔP*araE::frtfrt*P_CP18_*araE* Δ*rpoE*::*cat*	KMT414 x P1 (Δ*rpo*E::*cat*)
KMT473	MG1655 Δ*lacZYAfrtfrt lacI*^Q^ Δ*ara714* ΔP*araE::frtfrt*P_CP18_*araE* Δ*nsrR*::*tet* Δ*rseA*::*kan*	KMT470 x P1 (Δ*rseA*::*kan*)
KMT10003	MC1061 [Φl*rpoH* P3::*lacZ*] *rpoE*^+^ with suppressor of *rpoE*::WCm	CAG41001-lab collection
KMT12000	DJ480 *rybB*-*lacZ* long fusion (−69 to +22)	Thomspon et al. 2007 [15]
KMT12069	KMT12000 Δ*ara714 leu*::*Tn10*(*tet*)	Thomspon et al. 2007 [15]
KMT12078	KMT12069 Δ*rseA*::*kan*	Thomspon et al. 2007 [15]
KMT12090	KMT12078 pBAD24	Thomspon et al. 2007 [15]
KMT12091	KMT12078 pBAD-*nsrR*	Thomspon et al. 2007 [15]
KMT12093	KMT12000 Δl*ara714 leu*^+^	This work
KMT12094	KMT12093 Δ*rseA*::*kan*	This work
KMT12099	KMT12093 Δ*nsrR*::*tet*	This work
KMT12100	KMT12094 Δ*nsrR*::*tet*	This work
KMT12121	DJ480 *rybB*-*lacZ* [long fusion] Δ*ara714 leu*^+^ Δ*rseA*::*kan* Δ*nsrR*::*tet* pBAD24	12100 pBAD24 (TSS transformation)
KMT12122	DJ480 *rybB*-*lacZ* [long fusion] Δ*ara714 leu*^+^ Δ*rseA*::*kan* Δ*nsrR*::*tet* pBAD-*nsrR*	12100 pBAD-*nsrR* (TSS transformation)
KMT12123	DJ480 *rybB*-*lacZ* [long fusion] Δ*ara714 leu*^+^ Δ*rseA*::*kan* Δ*nsrR*::*tet* pBAD33	12100 pBAD33 (TSS transformation)
KMT12124	DJ480 *rybB*-*lacZ* [long fusion] Δ*ara714 leu*^+^ Δ*rseA*::*kan* Δ*nsrR*::*tet* pBAD33-*rseA*	12100 pBAD33-*rseA* (TSS transformation)
KMT12131	DJ480 *rybB*-*lacZ* [long fusion] Δ*ara714 leu*^+^ Δ*nsrR*::*tet* Δ*rpoE*::*kan*	KMT12130 x Δ*rpoE*::*kan* PCR kan^R^ at 37 °C on LB-kan after ara induction
KMT12132	DJ480 *rybB-lacZ* [long fusion] Δ*ara714 leu^+^* Δ*nsrR::tet* Δ*rpoE*::*kan* pBAD33 pTrc99A	KMT12131 + pBAD33 + pTrc99A (Electroporation and Amp^R^, Cm^R^)
KMT12133	DJ480 *rybB*-*lacZ* [long fusion] Δ*ara714 leu*^+^ Δ*nsrR*::*tet* Δ*rpoE*::*kan* pBAD33 + pCL245 (pTrc99A-*rpoE*)	KMT12131 + pBAD33 + pCL245 (pTrc99A-*rpoE*) (Electroporation and Amp^R^, Cm^R^)
KMT12134	DJ480 *rybB*-*lacZ* [long fusion] Δ*ara714 leu*^+^ Δ*nsrR*::*tet* Δ*rpoE*::*kan* pBAD33-*nsrR* + pCL245 (pTrc99A-rpoE)	KMT12131 + pBAD33-*nsrR* + pCL245 (pTrc99A-*rpoE*) (Electroporation and Amp^R^, Cm^R^)
KMT12218	MG1655 *lacI^q^ micA-lacZ*	NM580 x *micA*-*lacZ* PCR
KMT12294	MG1655 *lacI^q^ micA-lacZ* pBAD24	KMT12218 + pBAD24 (TSS Transformation)
KMT12295	MG1655 *lacI^q^ micA-lacZ* pBAD24-*nsrR*	KMT12218 + pBAD24-*nsrR* (TSS Transformation)
KMT14000	DJ480 Δ*ara714 leu*^+^ *rpoE270P2*-*lacZ* translational fusion	Thomspon et al. 2007 [15]
KMT14001	KMT14000 Δ*rseA*::*kan*	Thomspon et al. 2007 [15]
KMT14021	KMT14000 Δ*nsrR*::*tet*	This work
KMT14022	KMT14021 Δ*rseA*::*kan*	This work
KMT14029	DJ480 Δ*ara leu*+ Δ*nsrR*::*tet* Δ*rpoE*::*kan rpoE270*P2-*lacZ* translational fusion	KMT14021 x P1 (DJ480 Δ*rpoE*::*kan*)
KMT14031	DJ480 Δ*ara leu*+ Δ*nsrR*::*tet* Δ*rpoE*::*kan rpoE270*P2-*lacZ* translational fusion pTrc99A pBAD33	KMT14029 + pTrc99A & pBAD33 (double electroporation and selection on LB-Cm-Amp)
KMT14032	DJ480 Δ*ara leu*+ Δ*nsrR*::*tet* Δ*rpoE*::*kan rpoE270*P2-*lacZ* translational fusion pCL245 (pTrc99A-*rpoE*), pBAD33	KMT14029 + pCL245 (pTrc99A-*rpoE*) & pBAD33 (double electroporation and selection on LB-Cm-Amp)
KMT14033	DJ480 Δ*ara leu*+ Δ*nsrR*::*tet* Δ*rpoE*::*kan rpoE270*P2-*lacZ* translational fusion pCL245 (pTrc99A-*rpoE*), pBAD33-*nsrR*	KMT14029 + pCL245 (pTrc99A-*rpoE*) & pBAD33-*nsrR* (double electroporation and selection on LB-Cm-Amp)
NM200	DJ480 mini-l::*cat*	Nadim Majdalani, Ph.D.
NM300	DJ480 mini-l::*tet*	“ ”
Plasmid		
pBAD24	*araC*^+^, ColE1 *ori*, *bla*, MCS, Amp^R^	Guzman et al., 1995 [45]
pBAD33	*araC*^+^, p15A *ori*, *cat*, MCS, Cm^R^	“ ”
pBAD24-*nsrR*	*nsrR* gene cloned into *EcoR* I/*Pst* I site of MCS in pBAD24, Amp^R^	Thompson et al., 2007 [15]
pBAD33-*rseA*	*rseA* gene cloned into *Sac* I/*Xba* I of MCS in pBAD33, Cm^R^	This work
pCL245	*rpoE* gene cloned into pTrc99A, Amp^R^	Rhodius et al. 2006 [14]
pEB354 (T25)	Bacterial Adenylate Cylase Two Hybrid Interaction Expression Vector, P_Lac_, LacO site, CAP binding site, p15A ori, Adenylate Cyclase (*cya*) in T25 domain linker, (Kan^R^)	
pEB354-*zip* (T25-zip)	*zip* domain cloned into the *EcoR* I/*Xho* I site of pEB354, (Kan^R^)	This work
pEB354-*nsrR* (T25-*nsrR*)	*nsrR* gene cloned into the *EcoR* I/*Xho* I site of pEB354, (Kan^R^)	This work
pEB354-*zip* (T25-*rpoE*)	*rpoE* gene domain cloned into the *EcoR* I/*Xho* I site of pEB354, (Kan^R^)	This work
pEB355 (T18)	Bacterial Adenylate Cylase Two Hybrid Interaction Expression Vector, P_Lac_, LacO site, CAP binding site, ColE1 ori, Adenylate Cyclase (*cya*) in T18 domain linker, bla (Amp^R^)	
pEB355-*zip* (T8-zip)	*zip* domain cloned into the *EcoR* I/*Xho* I site of pEB355, *bla* (Amp^R^)	This work
pEB355-*zip* (T8-nsrR)	*nsrR* gene cloned into the *EcoR* I/*Xho* I site of pEB355, *bla* (Amp^R^)	This work
pEB355-*zip* (T8-rpoE)	*rpoE* gene cloned into the *EcoR* I/*Xho* I site of pEB355, *bla* (Amp^R^)	This work
pTrc99A	*lacI*^q^ ColE1 *ori*, *bla* MCS, IPTG inducible P*_trc_*	Thompson et al., 2007 [15]

**Table 3:** Oligonucleotides.

Oligonucleotide ID	Sequence 5′-3′	Purpose
KT75	tttctagattactgcgattgcgttcctaaagtttg	reverse primer for the amplification and cloning of *rseA* into pBAD33 or pBAD24 *Xba* I site
KT76	atgcagaaagaacaactttccgctttaatggatggcgaaacgctggatagaaagccacgttgtgtctcaa	Δ*rseA*::*kan* forward oligo for the amplification of recombineering substrate
KT77	ttactgcgattgcgttcctaaagtttgaattcctggcacctgtacagcgggcgctgaggtctgcctcgtg	Δ*rseA*::*kan* reverse oligo for the amplification of recombineering substrate
KT95	aagtccggagctcaggaggaattcaccatgcagaaagaacaactttccgctttaat	forward primer for the amplification and cloning of *rseA* into pBAD33 or pTrc99A *Sac* I site
KT237	ttatcatcaatataaatgtattttttcccgatttcccttttgaggttgatctagacatcattaattccta	forward primer for the amplification of Δ*nsrR*::*tet* recombineering substrate
KT238	tcttgtgacatctcggttcctccgttgtcatctctgatgaagattttcggaagctaaatcttctttatcg	reverse primer for the amplification of Δ*nsrR*::*tet* recombineering substrate
KT906	gcgtgagctcgatttcccttttgaggttgatgtgcag	forward primer for the amplification and cloning of *nsrR* into pBAD33 (21 nucleotides upstream of the ATG) *Sac* I site
KT907	gcgtctgcagtcactccaccagcaataatttataaag	reverse primer for the amplification and cloning of *nsrR* into pBAD33 *Pst* I site
KT926	TGGCGTTTCGATAGCGCGTGGAAATTTGGTTTGGGGAGACTTTACCTCGGgcgctgaggtctgcctcgtg	forward primer for Δ*rpoE*::*kan* recombineering substrate
KT927	AAGTTGTTCTTTCTGCATGCCTAATACCCTTATCCAGTATCCCGCTATCGaaagccacgttgtgtctcaa	reverse primer for the amplification of Δ*rpoE*::*kan* recombineering substrate

## Data Availability

The data are contained within the article.

## Supplementary Materials

The following supporting information can be downloaded at https://www.mdpi.com/article/10.3390/ijms26136318/s1.
