# Supplementary material for: NsrR Represses σE-Dependent Small RNAs and Interacts with RpoE via a Noncanonical Mechanism in Escherichia coli
Source: Int J Mol Sci. 2025 Jun 30;26(13):6318. doi: 10.3390/ijms26136318 (PMC12249550; doi:10.3390/ijms26136318)
Supplement: Supplementary file 1 [file ijms-26-06318-s001.zip › ijms-3716475-supplementary.pdf]

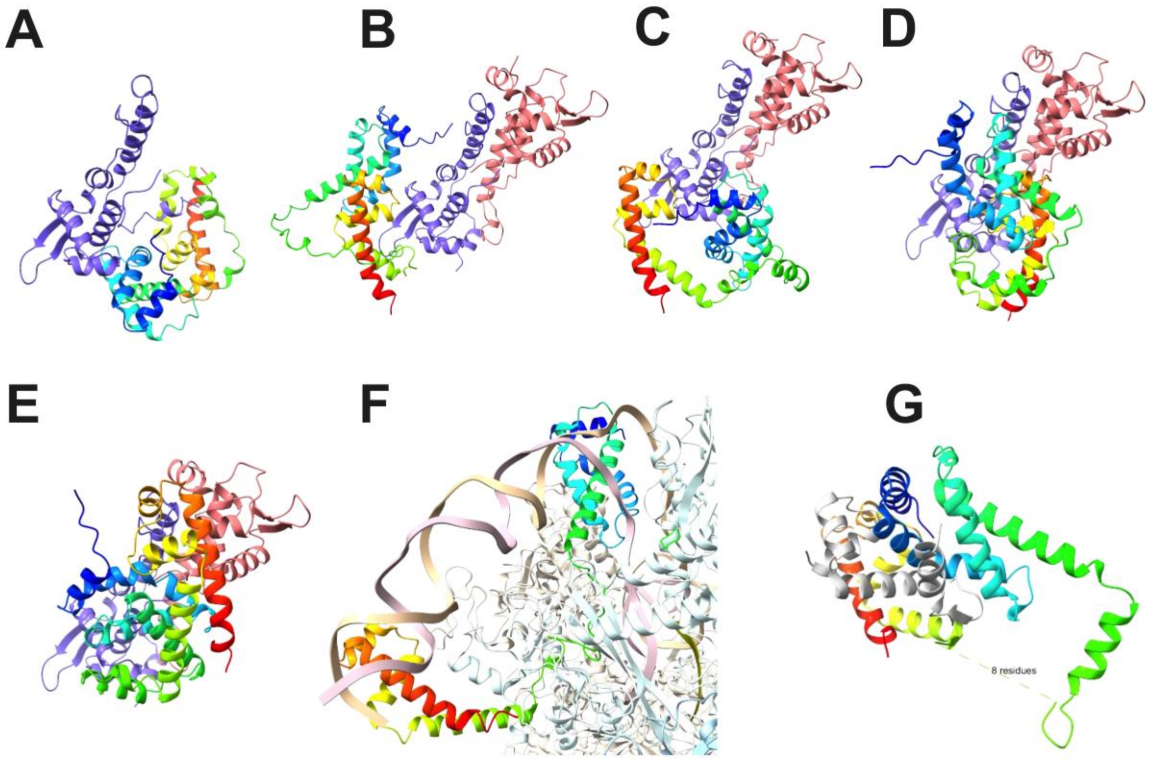

Supplemental Figure 1. (A) AF3 model of NsrR and RpoE heterodimer with pTM 0.48 and ipTM only 0.17 where the AF3 suggested cutoff are pTM 0.5 and ipTM 0.6. (B) to (E) the second to the 5th best model of NsrR dimer and RpoE. The coloring scheme is the same as in Figure 6D. Although the NsrR dimer is consistent, AF3 has difficulty to decide how they interact with RpoE; the pTM are between 0.54 and0.52 and ipTM are 0.4 or 0.39. (F) The *E. coli* RpoE transcription initiation complex with DNA and RNA, PDB ID 6JBQ. DNA is in baige and pale pink, 5 nt RNA in olive, RpoE in rainbow coloring, DNA-directed RNA polymerase subunit beta in light cyan and DNA-directed RNA polymerase subunit beta' in wheat color. (G) RpoE in complex with anti-sigma RseA, PDBID 1OR7. RpoE is in rainbow where 8 residues in the flexible loop region are missing, and RseA in light gray.
